# Antiphospholipid Syndrome and Catastrophic Antiphospholipid Syndrome: A Comprehensive Review of Pathogenesis, Clinical Features, and Management Strategies

**DOI:** 10.7759/cureus.66555

**Published:** 2024-08-10

**Authors:** Avinash Parepalli, Rajesh Sarode, Sunil Kumar, Manikanta Nelakuditi, M Jayanth Kumar

**Affiliations:** 1 Internal Medicine, Jawaharlal Nehru Medical College, Datta Meghe Institute of Higher Education and Research, Wardha, IND

**Keywords:** immunomodulatory therapy, pregnancy complications, antiphospholipid antibodies (apl), thrombosis, catastrophic antiphospholipid syndrome (caps), antiphospholipid syndrome (aps)

## Abstract

Antiphospholipid syndrome (APS) is an autoimmune disorder characterized by the presence of antiphospholipid antibodies (aPLs) that predispose individuals to thrombotic events and pregnancy-related complications. APS can occur as a primary condition or in association with other autoimmune diseases, most commonly systemic lupus erythematosus (SLE). Catastrophic APS (CAPS) is a rare, severe variant of APS, marked by rapid-onset, widespread thrombosis leading to multi-organ failure, often triggered by infections, surgical procedures, or cessation of anticoagulation therapy. Both APS and CAPS present significant clinical challenges due to their potential for severe morbidity and mortality. This comprehensive review aims to provide a detailed overview of the pathogenesis, clinical features, diagnostic criteria, and management strategies for APS and CAPS. The review highlights the immunological mechanisms underlying APS, including the role of aPLs, complement system activation, and endothelial cell dysfunction in developing thrombosis. It also outlines the clinical manifestations of APS, such as venous and arterial thrombosis, pregnancy morbidity, and neurological symptoms, along with the diagnostic criteria based on clinical and laboratory findings. The review delves into its pathogenesis, clinical presentation, and diagnostic challenges in the context of CAPS, emphasizing the need for immediate and intensive therapy to manage this life-threatening condition. Current management strategies for APS, including anticoagulant therapy, immunomodulatory treatments, and specific interventions for pregnancy-related complications, are discussed. The review highlights the importance of a multidisciplinary approach for CAPS, combining anticoagulation, high-dose corticosteroids, plasma exchange, and intravenous immunoglobulin. The review also addresses the prognosis and long-term outcomes for patients with APS and CAPS, underlining the necessity for ongoing monitoring and follow-up to prevent recurrent thrombotic events and manage chronic complications. Finally, future directions in research are explored, focusing on emerging therapies, biomarkers for early diagnosis, and the need for clinical trials to advance the understanding and treatment of these complex syndromes. By enhancing the understanding of APS and CAPS, this review aims to improve diagnosis, treatment, and patient care, ultimately leading to better health outcomes for those affected by these conditions.

## Introduction and background

Antiphospholipid syndrome (APS) is an autoimmune disorder characterized by the presence of antiphospholipid antibodies (aPLs) in the blood, which increase the risk of blood clots (thrombosis) in arteries and veins, as well as pregnancy-related complications such as recurrent miscarriages, stillbirths, and preterm deliveries [1]. APS can be a primary condition or secondary to other autoimmune diseases, most commonly systemic lupus erythematosus (SLE). The condition is diagnosed based on clinical criteria, including thrombotic events and pregnancy morbidity, in conjunction with laboratory tests that confirm the presence of aPL. APS represents a significant health burden due to its potential to cause chronic and life-threatening complications [2]. Catastrophic APS (CAPS), also known as Asherson's syndrome, is a rare but severe form of APS. CAPS is characterized by the rapid onset of widespread blood clots leading to multi-organ failure [3]. It is a medical emergency with high morbidity and mortality rates, requiring prompt diagnosis and aggressive treatment to improve patient outcomes. CAPS often presents following an infection, surgery, or cessation of anticoagulation therapy. The swift progression and complexity of CAPS make it a particularly challenging condition to manage [3].

Understanding APS and CAPS is crucial due to their significant impact on patient health and quality of life. APS is a major cause of morbidity, with recurrent thrombotic events potentially leading to chronic complications such as post-thrombotic syndrome, stroke, and pulmonary hypertension [4]. Pregnancy-related complications, including recurrent miscarriages and preterm deliveries, can result in emotional and physical distress for affected women, emphasizing the need for effective management strategies to ensure successful pregnancies. Moreover, the economic burden associated with the long-term management of APS is substantial, further highlighting the importance of comprehensive care [4]. CAPS, although rare, represents a medical emergency with high fatality rates if not promptly and effectively treated. The rapid progression and complexity of CAPS require a multidisciplinary approach to manage the acute phase and prevent long-term sequelae [5]. Increasing awareness and understanding of these conditions among healthcare professionals can lead to earlier diagnosis, improved management, and better patient outcomes. Additionally, enhancing public and patient awareness about APS and CAPS can promote early symptom recognition and timely medical intervention [5].

The primary objectives of this comprehensive review are to provide a detailed overview of the pathogenesis of APS and CAPS, highlighting the underlying mechanisms and contributing factors. This review aims to describe the clinical features and diagnostic criteria of APS and CAPS, enabling better recognition and differentiation of these conditions. Furthermore, it seeks to review current management strategies for APS and CAPS, including anticoagulant therapy, immunomodulatory treatments, and specific interventions for pregnancy-related complications. The review will also discuss the prognosis and long-term outcomes for patients with APS and CAPS, emphasizing the importance of ongoing monitoring and follow-up. Finally, this review will explore future directions and research opportunities, including emerging therapies and potential biomarkers for early diagnosis and prognosis. By addressing these objectives, this review aims to enhance the understanding of APS and CAPS among clinicians, researchers, and patients, ultimately contributing to improved diagnosis, treatment, and patient care.

## Review

Pathogenesis of APS

Etiology and Risk Factors

**Figure 1:**
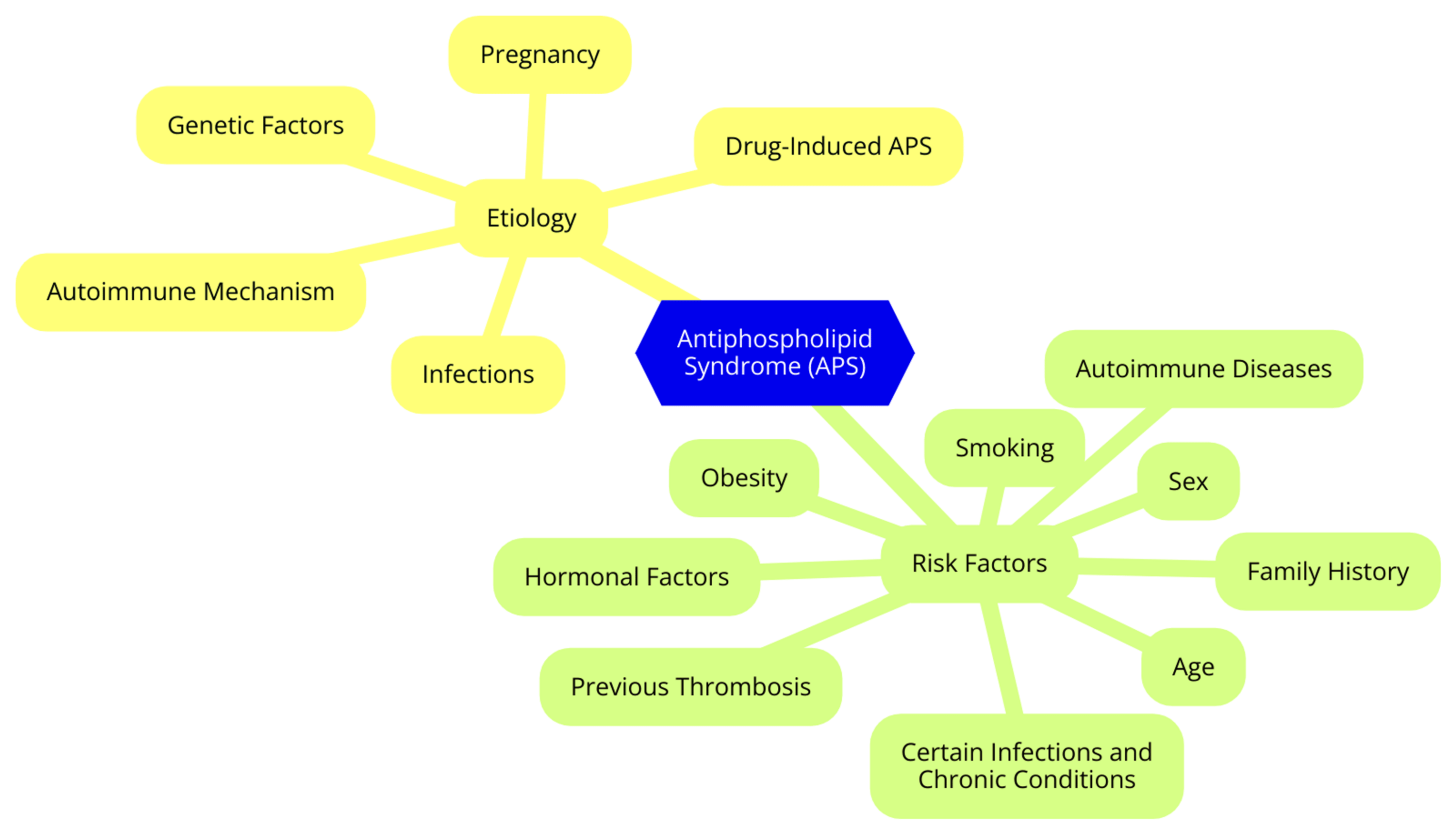
Etiology and risk factors of antiphospholipid syndrome Image Credit: Dr. Avinash Parepalli

The etiology and risk factors for APS can be broadly categorized into genetic factors and environmental triggers. Certain human leukocyte antigen (HLA) alleles, such as HLA-DR4, HLA-DR7, and HLA-DRw53, have been associated with an increased risk of developing APS, suggesting that immune dysregulation plays a role in the disease's pathogenesis [6]. Family studies have revealed a higher incidence of aPLs and related clinical manifestations among first-degree relatives of APS patients, indicating a potential genetic predisposition. However, the exact inheritance patterns remain undefined [6]. Infections, particularly viral infections, have been identified as potential triggers for developing aPLs and the onset of APS. Pathogens such as cytomegalovirus, Epstein-Barr virus, and parvovirus B19 have been associated with inducing aPL production through molecular mimicry mechanisms [7]. Certain medications, including hydralazine, procainamide, and quinidine, have been linked to the development of aPLs and, in some cases, APS. Cancer and its treatments, such as chemotherapy and radiation therapy, have also been associated with an increased risk of developing aPLs. However, the direct link between malignancies and the onset of clinical APS remains less clear. Pregnancy itself can be considered an environmental trigger for APS, as the physiological changes during gestation may unmask or exacerbate the condition. The postpartum period is also a time of increased risk for thrombotic events in women with APS [8]. The etiology and risk factors of antiphospholipid syndrome are shown in Figure 1.

Immunological Mechanisms

The pathogenesis of APS involves a complex interplay of immunological mechanisms, with aPLs playing a central role. These autoantibodies primarily target β2-glycoprotein I (β2GPI) and anionic phospholipids on cell membranes. Their presence is essential for the development of APS [9]. However, aPL typically requires an additional trigger, often an inflammatory event, to induce thrombosis. The procoagulant effects of aPL include the formation of phospholipid-protein complexes and direct impacts on trophoblasts during pregnancy, leading to placental dysfunction and complications such as recurrent miscarriage [9]. The complement system also significantly contributes to the pathogenesis of APS. Dysregulation of this system can lead to uncontrolled activation, exacerbating thrombosis and tissue injury [10]. Evidence suggests that aPL can activate the complement cascade, intensifying the inflammatory response and causing endothelial damage. This activation is especially relevant in pregnancy-related complications, where complement-mediated injury can contribute to fetal loss [10]. Endothelial cell dysfunction is another critical factor in APS-related thrombosis. aPL can directly affect endothelial cells, inducing a procoagulant state [11]. This includes increased expression of adhesion molecules, which enhances the binding of platelets and leukocytes to the endothelium, promoting thrombus formation. Additionally, endothelial cells may reduce the secretion of natural anticoagulants, further shifting the balance toward thrombosis [11]. Platelet activation is also crucial in the pathogenesis of APS. aPL can increase platelet reactivity, contributing to the thrombotic events characteristic of the syndrome [12]. aPL induces platelet activation by directly binding to platelets or indirectly activating endothelial cells, which release pro-aggregatory substances. Activated platelets release procoagulant factors, forming stable thrombi in both venous and arterial systems [12].

Pathophysiology of Thrombosis

The pathophysiology of thrombosis can be classified into three primary types: venous thrombosis, arterial thrombosis, and microthrombosis, each with distinct mechanisms and contributing factors. Venous thrombosis typically occurs in the deep veins of the legs and is often associated with conditions that promote venous stasis, endothelial injury, and hypercoagulability-collectively known as Virchow's triad [13]. Venous stasis, caused by prolonged immobility, obesity, or heart failure, increases blood viscosity. Endothelial injury from trauma or surgery exposes subendothelial collagen, facilitating platelet adhesion and activation. Hypercoagulability, which can be inherited or acquired, is influenced by conditions such as cancer, pregnancy, and hormonal therapies, all of which elevate thrombus formation risk. Complications of venous thrombosis include pulmonary embolism (PE), where a dislodged thrombus travels to the pulmonary arteries, potentially leading to fatal outcomes [13]. Arterial thrombosis commonly arises in the context of atherosclerosis, leading to thrombus formation in arteries and potentially resulting in myocardial infarction or stroke. This process begins with endothelial dysfunction due to atherosclerosis, marked by lipid accumulation and inflammation that expose pro-coagulant factors and promote platelet activation. Endothelial injury triggers platelet adhesion to the damaged site, followed by platelet activation and aggregation to form a thrombus [14]. Conditions, such as hypertension and diabetes, exacerbate this process by increasing platelet reactivity, activating the coagulation cascade, converting fibrinogen to fibrin, and stabilizing the thrombus. Arterial thrombosis can cause acute ischemic events like heart attacks and strokes, requiring prompt medical intervention [14]. Microthrombosis involves the formation of small thrombi within the microcirculation and is often associated with conditions such as sepsis, disseminated intravascular coagulation (DIC), and certain autoimmune disorders. Inflammatory cytokines and mediators activate endothelial cells, increasing the expression of adhesion molecules and pro-coagulant factors that facilitate microthrombus formation [15]. Altered blood flow in the microcirculation can predispose individuals to microthrombus formation, as seen in septic patients where sepsis-induced coagulopathy leads to widespread microvascular thrombosis. Hypercoagulable states, similar to those observed in venous and arterial thrombosis, can also contribute to microthrombosis by creating an imbalance between pro-coagulant and anticoagulant factors. The clinical implications of microthrombosis include organ dysfunction and failure due to impaired blood flow and oxygen delivery, further complicating the morbidity associated with severe infections and inflammatory conditions. Understanding these various forms of thrombosis is crucial for developing effective prevention and treatment strategies [15].

Clinical features of APS

Primary vs. Secondary APS

APS can be divided into two primary types: primary APS (PAPS) and secondary APS (SAPS). Primary APS occurs in individuals without any associated autoimmune disease and is characterized by the presence of aPLs along with clinical manifestations such as thrombosis and pregnancy complications. Patients with PAPS may experience both venous and arterial thrombosis, as well as obstetric issues like recurrent miscarriages and preeclampsia [16]. This form of APS is more common in women, with a male-to-female ratio of approximately 1:3.5. In contrast, secondary APS occurs in patients with underlying autoimmune conditions, most frequently SLE. While SAPS shares similar thrombotic and obstetric complications with PAPS, patients may also present with additional features related to their autoimmune disease, such as autoimmune hemolytic anemia and endocardial valve disease [17]. The male-to-female ratio in SAPS is more pronounced, approximately 1:7, reflecting the higher incidence of SLE in women. Key differences between the two types include their association with autoimmune diseases, clinical manifestations, laboratory findings, and the long-term evolution of the conditions. Notably, patients with PAPS generally do not have anti-dsDNA or anti-extractable nuclear antigen antibodies, indicative of SLE in SAPS. Understanding these distinctions is crucial for tailoring treatment strategies and monitoring potential complications in patients with APS [18].

Common Clinical Manifestations

APS is associated with a range of common clinical manifestations, primarily involving thrombosis, pregnancy complications, hematological abnormalities, and neurological issues [19]. Thrombosis, both venous and arterial, is a hallmark of APS. Deep vein thrombosis (DVT) is the most frequent manifestation, typically occurring in the lower extremities and potentially leading to PE. This serious complication can cause chest pain, shortness of breath, and life-threatening consequences. Arterial thrombosis in APS can result in ischemic strokes, transient ischemic attacks (TIAs), myocardial infarctions, and thrombosis in atypical locations such as the renal veins, mesenteric arteries, and upper extremities [19]. Pregnancy complications are another significant feature of APS, especially in women with aPLs. Recurrent pregnancy loss, often due to placental insufficiency and thrombotic events affecting placental blood flow, is common [20]. Additionally, preeclampsia-a pregnancy-related condition characterized by high blood pressure and potential organ dysfunction - and intrauterine growth restriction (IUGR) - resulting from reduced blood flow to the placenta) can also occur in women with APS [20]. Hematological abnormalities, such as thrombocytopenia (low platelet count), are observed in approximately 15% of patients with APS. While most cases are mild and do not pose significant bleeding risks, severe thrombocytopenia can occasionally lead to complications, particularly during surgical procedures or trauma. Neurological complications in APS can be diverse, including ischemic events like strokes and TIAs, cognitive dysfunction, migraines, and, in rare instances, seizures [21].

Diagnostic Criteria

The diagnosis of APS is based on a combination of clinical and laboratory criteria, as outlined by the Sydney classification. This classification requires the presence of at least one clinical criterion and one laboratory criterion to confirm the diagnosis of APS [22]. Laboratory tests are essential for identifying aPLs, central to APS pathogenesis. Key tests include detecting lupus anticoagulant (LA), anticardiolipin antibodies (aCLs), and anti-β2 glycoprotein I antibodies (aβ2GPIs) [23]. LA, a type of aPL, disrupts normal coagulation processes, resulting in a prolonged activated partial thromboplastin time (aPTT) in vitro. A positive test result must be confirmed on two separate occasions for a definitive diagnosis of LA, with a minimum interval of 12 weeks. Common methods for testing LA include dilute Russell's viper venom time (dRVVT) and aPTT [24]. Anticardiolipin antibodies are another critical diagnostic component. These antibodies target cardiolipin, a phospholipid present in cell membranes. APS diagnosis requires the presence of IgG or IgM isotypes of aCL at medium to high titers (≥40 GPL or MPL units) on two separate occasions, spaced at least 12 weeks apart [23]. Similarly, aβ2GPI target β2-glycoprotein I, a protein crucial to the coagulation cascade. For a diagnosis, the presence of IgG or IgM isotypes of these antibodies at moderate to high titers (≥99th percentile) must be confirmed on two separate occasions [23]. In addition to laboratory criteria, clinical features are vital for diagnosing APS. The clinical criteria focus on thrombotic events and obstetric complications. At least one of these manifestations must be present for diagnosis [25]. Thrombotic events include venous thrombosis, such as DVT or PE, and arterial thrombosis, which may present as strokes, myocardial infarctions, TIAs, or thrombosis in atypical locations like renal veins or mesenteric vessels [25]. Obstetric complications also form a critical aspect of the clinical criteria for APS. Women with APS may experience recurrent pregnancy loss, defined as three or more consecutive spontaneous miscarriages before ten weeks of gestation or one or more fetal deaths after ten weeks. Additionally, premature birth before 34 weeks due to severe preeclampsia or placental insufficiency is considered a significant clinical criterion [26].

Catastrophic APS (CAPS)

Definition and Overview

APS is an autoimmune disorder characterized by the presence of aPLs, which markedly increase the risk of thrombosis (blood clots) and pregnancy complications. These antibodies target membrane phospholipids and phospholipid-binding proteins, disrupting normal coagulation processes and leading to a hypercoagulable state [27,28]. Clinically, APS manifests as recurrent venous or arterial thrombosis, such as DVT, PE, stroke, and myocardial infarction. It also presents with pregnancy-related issues, including recurrent miscarriages, preeclampsia, and placental insufficiency. The syndrome predominantly affects women, particularly those aged 30 to 40. It is often associated with other autoimmune disorders, notably SLE, in which aPLs are present in 30%-40% of patients [20]. Diagnosis of APS relies on clinical criteria and laboratory tests that detect aPLs. Key tests include the detection of LAs, aCLs, and anti-beta-2 glycoprotein I antibodies. For a definitive diagnosis, at least one clinical event (thrombosis or a pregnancy complication) and one laboratory test must be confirmed twice. Management of APS is individualized and generally involves anticoagulation therapy for patients with a history of thrombosis, low-dose aspirin for prophylaxis, and close monitoring for pregnant women to minimize risks to both mother and fetus. Addressing underlying autoimmune disorders is also crucial for effective management [23].

Pathogenesis of CAPS

CAPS is a rare and severe form of APS characterized by rapid and widespread thrombosis that leads to multiorgan failure. The pathogenesis of CAPS involves a combination of identifiable triggers and complex immunological mechanisms. Approximately 49% of CAPS cases are associated with respiratory, urinary tract, and gastrointestinal infections, which can trigger complement activation and subsequent thrombotic events. Surgery or significant physical trauma, which accounts for about 17% of cases, can also precipitate CAPS. Certain cancers, especially hematologic malignancies like lymphomas and leukemias, as well as solid tumors (e.g., lung or colon adenocarcinoma), are linked to about 16% of CAPS cases. Discontinuation or inadequate dosing of anticoagulant therapy can trigger CAPS in about 8% of patients. At the same time, pregnancy-related events, such as HELLP syndrome and placental infarction, can also be precipitating factors, occurring in about 8% of cases [29]. These factors often act as a “second hit” in individuals with underlying aPLs, leading to the clinical manifestations of CAPS. aPLs are central to the pathogenesis of CAPS, activating endothelial cells, platelets, and monocytes, creating a proinflammatory and prothrombotic environment. CAPS is also associated with a vicious complement activation cycle, contributing to microvascular thrombosis and tissue damage. The inflammatory response in CAPS can lead to a cytokine storm characterized by the release of pro-inflammatory cytokines that further enhance coagulation and promote vascular injury [30]. Elevated levels of von Willebrand factor and P-selectin in circulation indicate significant endothelial and platelet activation, which are hallmarks of the thrombotic process in CAPS. Understanding the complex interplay of these triggers and mechanisms is crucial for recognizing and managing CAPS effectively [31].

Clinical Features

CAPS is a severe and life-threatening condition characterized by rapidly progressive multiorgan failure and thrombosis across multiple organ systems. The onset of multiorgan failure typically occurs within a week and is often accompanied by a systemic inflammatory response due to extensive tissue damage. Commonly affected organs include the kidneys, which may present with acute renal failure, hypertension, proteinuria, and hematuria [32]. The lungs can be compromised, leading to complications such as acute respiratory distress syndrome (ARDS). The central nervous system may exhibit various neurological symptoms, including headaches, seizures, and altered mental status. Cardiac involvement can result in heart failure or arrhythmias, and cutaneous manifestations, such as livedo reticularis and digital gangrene, may offer early diagnostic clues. Thrombosis is a defining feature of CAPS, characterized by disseminated intravascular thrombosis that leads to ischemia and infarction in various organs [33]. Common sites of thrombosis include the renal and pulmonary vasculature, causing renal impairment and PE, respectively. Involvement of the cerebral and cardiac vasculature can result in strokes and myocardial infarctions. Histologically, CAPS is marked by acute thrombotic microangiopathy, with tissue samples revealing small-vessel occlusion and ischemic damage. Laboratory findings may include thrombocytopenia and mild microangiopathic hemolytic anemia, although severe thrombocytopenia is less common. Recognizing these clinical features is crucial for clinicians to initiate timely management strategies and improve patient outcomes in this rapidly progressing syndrome [34].

Diagnostic Criteria

The clinical diagnosis of CAPS requires evidence of involvement of three or more organs, systems, or tissues. This multiorgan involvement is a defining syndrome feature and can present in various ways. Renal involvement may manifest as acute renal failure or renal infarction, while pulmonary complications can include ARDS or PE [35]. Neurological symptoms may range from strokes to TIAs, and cardiac manifestations can encompass myocardial infarction or valvular issues. Other systems, such as the skin, gastrointestinal tract, and adrenal glands, may also be affected, leading to conditions like livedo reticularis, bowel infarction, or adrenal insufficiency. The rapid onset of these symptoms, typically within a week, is a critical aspect of the diagnosis [35]. In addition to clinical criteria, laboratory tests are essential for diagnosing CAPS. aPLs must be confirmed at least two times, spaced at least six weeks apart. Key laboratory tests include the detection of LA, anticardiolipin antibodies (aCL) of both IgG and IgM isotypes at medium or high titers, and aβ2GPIs of IgG and IgM isotypes in medium or high titers [1]. These tests are crucial for establishing the autoimmune basis of the syndrome. Furthermore, histological confirmation of small-vessel occlusion in at least one organ or tissue is necessary to complete the diagnostic picture [1].

Management Strategies for APS

APS requires a comprehensive management strategy to mitigate the risk of thrombotic events and address associated complications. This strategy encompasses several key areas, including general management principles, antithrombotic therapy, management of pregnancy complications, and immunomodulatory therapies [36]. A fundamental component of APS management is risk factor modification. Patients are advised to adopt a healthy lifestyle, incorporating regular physical activity, a balanced diet, weight management, and smoking cessation [37]. Additionally, controlling comorbidities such as hypertension, diabetes, and hyperlipidemia is crucial to reducing overall cardiovascular risk. Regular monitoring and follow-up are essential to assess thrombotic risk and evaluate the effectiveness of anticoagulation therapy. Routine blood tests to check INR levels are necessary for patients on warfarin. Educating patients about recognizing thrombosis symptoms and the importance of adherence to prescribed treatments can enhance their involvement in their care [37]. Antithrombotic therapy is central to APS management. Anticoagulants are typically the first line of defense against thrombotic events. In acute settings or during pregnancy, heparin, particularly low molecular weight heparin (LMWH), is preferred due to its favorable safety profile. Warfarin is commonly used for long-term anticoagulation in most APS patients, with a target INR of 2.0 to 3.0 [38]. Direct oral anticoagulants (DOACs) are emerging as potential alternatives, especially for those with a history of venous thrombosis. However, more research is needed to confirm their safety and efficacy in APS. Additionally, antiplatelet agents like low-dose aspirin are often employed, particularly in patients with a history of arterial thrombosis or during pregnancy, to further mitigate the risk of complications [38].

Effective management of pregnancy complications in women with APS is crucial for improving outcomes. Prophylactic anticoagulation with LMWH is typically started before conception or as soon as pregnancy is confirmed and continued throughout pregnancy and postpartum to minimize the risk of miscarriage and other complications [39]. Alongside LMWH, low-dose aspirin is commonly used to further reduce the risk of pregnancy-related issues, especially in women with a history of recurrent pregnancy loss. This combined approach has enhanced pregnancy outcomes for affected individuals [39]. Immunomodulatory therapies are important, particularly for patients with secondary APS associated with other autoimmune conditions. Hydroxychloroquine can be beneficial by potentially reducing thrombotic risk and improving overall disease management [40]. Corticosteroids may be indicated for patients with severe manifestations of APS or those with associated autoimmune diseases, helping to control inflammation and modulate the immune response. Intravenous immunoglobulin (IVIG) is considered for cases of refractory APS or severe manifestations, as it may reduce antibody levels and modulate the immune system. Additionally, rituximab, a B-cell-depleting agent, may be used in severe or refractory APS cases, especially with concurrent autoimmune disorders. Other biologics are under investigation for their potential role in APS management [40].

Management Strategies for CAPS

The management of CAPS demands a comprehensive and aggressive approach due to its rapid progression and high morbidity and mortality rates. Effective treatment requires immediate, intensive therapy, adjunctive interventions, and a multidisciplinary approach to optimize patient outcomes [41]. Anticoagulation remains the cornerstone of CAPS treatment. Initial therapy typically involves administering unfractionated heparin or low-molecular-weight heparin (LMWH) to prevent further thrombotic events [42]. Once stabilized, patients are transitioned to long-term anticoagulation with warfarin, aiming for an international normalized ratio (INR) target of 2.0-3.0. In some cases, DOACs may be considered, though evidence supporting their use in CAPS is still limited [42]. High-dose corticosteroids are critical for managing CAPS, as they rapidly reduce inflammation and immune-mediated damage. Methylprednisolone is commonly used, starting at 1-2 mg/kg/day and adjusted based on clinical response. Plasma exchange (plasmapheresis) is also employed to remove circulating aPLs and inflammatory mediators, helping to restore normal hemostasis [43]. Plasma exchange is typically performed daily or every other day, depending on the severity of the condition and patient response. Additionally, IVIG can modulate immune responses and inhibit complement activation, providing further support. IVIG is usually given at a dose of 1 g/kg over two days or according to clinical guidelines [43].

For patients who do not respond adequately to initial therapies, cyclophosphamide may be indicated, particularly for those with CAPS associated with SLE. This immunosuppressive agent helps control the underlying autoimmune process and is typically administered orally or intravenously, often in a pulse regimen (e.g., 500-1,000 mg/m² every one to three months) [44]. Emerging therapies and biologics are also gaining attention in CAPS management. Rituximab, a monoclonal antibody targeting CD20, has shown efficacy in refractory cases and patients with SLE. Eculizumab, a complement inhibitor, has demonstrated promise in severe, therapy-resistant CAPS cases. Ongoing research continues to explore additional biologics and targeted therapies to enhance outcomes in this complex syndrome [45]. Given the complexity of CAPS, a multidisciplinary approach is crucial. Most patients require admission to an intensive care unit (ICU) for close monitoring and management due to the risk of multiorgan failure. Coordination among critical care specialists, rheumatologists, and hematologists is essential for optimal care [46]. Organ support and replacement therapies may be necessary for patients with complications. Dialysis may be required for renal failure, with continuous renal replacement therapy (CRRT) often preferred in critically ill patients. Mechanical ventilation may be needed for those with respiratory failure from PE or other complications. Vasopressors may be employed to manage hypotension and maintain organ perfusion in cases of septic shock or severe hypotension [47].

Prognosis and Long-Term Outcomes

The risk of thrombotic recurrence in patients with APS is a significant concern. Studies indicate that approximately 25% to 44% of patients experience a new thrombotic event within four to 10 years of follow-up. A cohort study with a median follow-up of about 172.5 months found a recurrence rate of 40.2% among patients with persistent aPLs [1]. Several factors contribute to this risk, including the specific aPL profile. Patients with multiple positive aPLs are at higher risk, particularly those with IgG anti-β2-GPI and IgG aCL positivity. Additionally, the nature of the initial thrombotic event is crucial; for example, patients who initially present with arterial thrombosis are more likely to experience subsequent arterial recurrences. Compliance with anticoagulation therapy is also critical, as non-compliance can lead to increased rates of thrombotic events [48]. Pregnancy outcomes for women with APS have improved significantly with appropriate management. Current data suggest that women with APS have a 70% chance of achieving a successful pregnancy when treated with low-dose aspirin and heparin, a notable increase from the 20% success rate observed in untreated cases [49]. This improvement underscores the importance of targeted therapy in managing pregnancy complications associated with APS, such as recurrent miscarriages and preeclampsia. With proper medical care, many women with APS can now anticipate positive pregnancy experiences and outcomes [49]. CAPS is a rare but severe APS variant characterized by rapid-onset multi-organ failure due to widespread thrombosis. This condition carries a high mortality rate, estimated at around 30%, even with aggressive treatment. The morbidity associated with CAPS is also significant, as many survivors face severe complications that can lead to long-term disability. The rapid progression of CAPS often results in acute organ damage, necessitating immediate and intensive medical intervention to mitigate the effects of the widespread thrombotic events [41]. Long-term organ damage is a common consequence of CAPS, as extensive thrombosis can severely impact various organ systems. Survivors may experience renal impairment, with acute kidney injury being prevalent, and many may develop chronic kidney disease as a result of prior thrombotic episodes [50]. Neurological deficits are another concern, as thrombosis affecting cerebral circulation can lead to lasting cognitive impairments and motor dysfunction. Additionally, pulmonary complications, such as pulmonary hypertension and other respiratory issues, may arise due to vascular damage from thrombotic events [50].

Future directions and research

Future research in APS and CAPS focuses on several key areas to enhance the diagnosis, treatment, and understanding of these complex disorders. One significant area of exploration is the identification of emerging biomarkers to improve the diagnosis and monitoring of APS. Developing specific biomarkers could enable better stratification of patients based on their thrombotic risk and guide personalized treatment strategies [51]. In addition to biomarkers, research is ongoing into novel therapeutic approaches, especially for managing cardiovascular complications associated with APS. Potential therapies under investigation include biologics that target the immune response, such as B-cell-directed therapies and other immunomodulators. Complement inhibition is another promising area, given its role in the pathophysiology of APS. Therapies targeting the complement system may offer new treatment options, particularly for severe cases like CAPS. Researchers are also evaluating the efficacy of triple therapy, which combines anticoagulants, corticosteroids, and immunomodulatory agents, for managing CAPS to improve outcomes and reduce mortality rates [52]. Understanding the underlying mechanisms of APS and CAPS remains critical for future research. Further studies are needed to clarify the relationship between aPLs and thrombotic events and to investigate the role of environmental triggers such as infections and trauma in precipitating CAPS. Insights into these mechanisms could lead to targeted interventions and preventive strategies, ultimately enhancing patient care [1]. As knowledge about APS and CAPS progresses, there is a pressing need for updated clinical guidelines that incorporate the latest research findings. This includes recommendations for managing cardiovascular risks in older adults with APS and strategies for addressing pregnancy-related complications. Integrating the most recent research into clinical practice will enable healthcare providers to offer more effective and tailored management for patients with these complex syndromes [53].

## Conclusions

In conclusion, APS and CAPS are complex autoimmune disorders with profound clinical implications. APS, with its varied manifestations, significantly impacts morbidity and quality of life, while CAPS, though rare, poses a severe and life-threatening challenge requiring urgent and aggressive treatment. This review has highlighted the critical aspects of their pathogenesis, clinical features, and management strategies, underscoring the necessity for early diagnosis and tailored therapeutic approaches. Advances in understanding the underlying mechanisms and the development of novel therapies hold promise for improving outcomes. Continued research and collaboration across disciplines are essential to uncover new insights, enhance diagnostic accuracy, and refine treatment protocols, ultimately aiming to reduce morbidity, prevent complications, and improve the prognosis for patients with APS and CAPS. We can strive towards better patient care and outcomes in these challenging conditions by fostering greater awareness and advancing clinical practices.
